# Mutational analysis and protein profiling predict drug sensitivity in multiple myeloma cell lines

**DOI:** 10.3389/fonc.2022.1040730

**Published:** 2022-11-29

**Authors:** Mariaserena Giliberto, Leonardo Miranda Santana, Toril Holien, Kristine Misund, Sigve Nakken, Daniel Vodak, Eivind Hovig, Leonardo A. Meza-Zepeda, Eivind Coward, Anders Waage, Kjetil Taskén, Sigrid S. Skånland

**Affiliations:** ^1^ Department of Cancer Immunology, Institute for Cancer Research, Oslo University Hospital, Oslo, Norway; ^2^ K.G. Jebsen Centre for B Cell Malignancies, Institute of Clinical Medicine, University of Oslo, Oslo, Norway; ^3^ Oslo Centre for Biostatistics and Epidemiology, University of Oslo, Oslo, Norway; ^4^ Department of Clinical and Molecular Medicine, Norwegian University of Science and Technology, Trondheim, Norway; ^5^ Department of Hematology, St. Olav’s University Hospital, Trondheim, Norway; ^6^ Department of Immunology and Transfusion Medicine, St. Olav’s University Hospital, Trondheim, Norway; ^7^ Norwegian Cancer Genomics Consortium, Oslo University Hospital, Oslo, Norway; ^8^ Department of Tumor Biology, Institute for Cancer Research, Oslo University Hospital, Oslo, Norway; ^9^ Centre for Cancer Cell Reprogramming, Institute of Clinical Medicine, Faculty of Medicine, University of Oslo, Oslo, Norway; ^10^ Genomics Core Facility, Department of Core Facilities, Institute for Cancer Research, Oslo University Hospital, Oslo, Norway; ^11^ Center for Bioinformatics, Department of Informatics, University of Oslo, Oslo, Norway; ^12^ Bioinformatics Core Facility, Norwegian University of Science and Technology, Trondheim, Norway

**Keywords:** drug sensitivity screening, multiple myeloma, mutations, targeted therapy, MEK, PI3K, drug response biomarkers, precision medicine

## Abstract

**Introduction:**

Multiple myeloma (MM) is a heterogeneous disease where cancer-driver mutations and aberrant signaling may lead to disease progression and drug resistance. Drug responses vary greatly, and there is an unmet need for biomarkers that can guide precision cancer medicine in this disease.

**Methods:**

To identify potential predictors of drug sensitivity, we applied integrated data from drug sensitivity screening, mutational analysis and functional signaling pathway profiling in 9 cell line models of MM. We studied the sensitivity to 33 targeted drugs and their association with the mutational status of cancer-driver genes and activity level of signaling proteins.

**Results:**

We found that sensitivity to mitogen-activated protein kinase kinase 1 (MEK1) and phosphatidylinositol-3 kinase (PI3K) inhibitors correlated with mutations in *NRAS/KRAS*, and *PI3K* family genes, respectively. Phosphorylation status of MEK1 and protein kinase B (AKT) correlated with sensitivity to MEK and PI3K inhibition, respectively. In addition, we found that enhanced phosphorylation of proteins, including Tank-binding kinase 1 (TBK1), as well as high expression of B cell lymphoma 2 (Bcl-2), correlated with low sensitivity to MEK inhibitors.

**Discussion:**

Taken together, this study shows that mutational status and signaling protein profiling might be used in further studies to predict drug sensitivities and identify resistance markers in MM.

## Introduction

MM is considered a treatable, but generally incurable disease, with a heterogeneous clinical course as one of its hallmarks. Several types of molecular alterations, such as single-nucleotide variants and cytogenetic abnormalities, are responsible for disease initiation, maintenance, and progression (1). Advances in molecular profiling technologies have enabled us to characterize the molecular landscape of aberrations, which in turn improves our understanding of the underlying cancer biology, and indicates potential molecular treatment targets.

Many MM driver mutations occur in known cancer-signaling pathways, such as the Rat sarcoma virus (RAS), mitogen-activated protein kinase (MAPK), and PI3K/AKT pathways, which are known to control proliferation and survival of MM cells (2, 3). Sequencing analysis of MM patient samples has shown that the Kirsten RAS (*KRAS*) oncogene is the most commonly mutated gene (36%) in the disease, followed by the neuroblastoma RAS (*NRAS*) (20%), with frequent co-existence of one or more variants in both KRAS and NRAS cancer-driver genes (4–6). Interestingly, refractory MM patients with multi-drug resistance to standard myeloma therapies (e.g. proteasome inhibitors and immunomodulatory drugs) showed a significant increase (72%) in the mutation rate of RAS pathway genes, as compared to newly diagnosed MM cases (7). This suggests that RAS mutations play a role in acquired drug resistance in refractory MM. Targeting the RAS signaling pathway is therefore of potentially high therapeutic interest.

Studies in MM have shown that molecular features, including mutations, translocations and copy-number abnormalities, have both prognostic and predictive value and may enable further improvement in patient outcomes if employed to define personalized treatment strategies (8). Inhibition of RAS effectors, such as MEK, has emerged as a viable strategy for the treatment of KRAS/NRAS mutant MM clones (9, 10). Specifically, treatment of RAS/RAF mutant MM with the MEK inhibitor trametinib has shown good tolerability and durable remission in some patients (9, 11).

Besides the RAS signaling pathway, the PI3K/AKT/mTOR pathway is activated in a significant proportion of MM patients (3). Increased levels of MM-promoting cytokines, including interleukin-6 (IL6) and insulin-like growth factor-1 (IGF1), have been reported to be involved in the activation of this pathway as well as the aberrant up-regulation of other pathways that feed into the PI3K/AKT activation complex (12). Inhibition of PI3K/AKT/mTOR pathway induces apoptosis in MM (13), and targeting this pathway may therefore show therapeutic benefit.

MM mutational status is currently used to guide the use of targeted agents in precision medicine trials, including the MyDrug study (NCT03732703), the CAPTUR study (NCT03297606), and the MATCH screening trial (NCT02465060). Protein expression and phosphorylation levels also impact drug responsiveness, arguing for integrating both genetic and functional analyses in treatment selection strategies (14–16).

Here, we used high-throughput DNA sequencing to characterize the genetic make-up of nine MM cell lines. Based on the spectrum of mutated targets and their druggability, we designed a library of 33 targeted drugs, which was used on the same MM cell lines. Associations between mutational status and drug sensitivity were investigated. Next, we used a dataset of signaling readouts (n=31) available on the same cell lines (16) to study how drug sensitivity correlated with baseline protein expression and phosphorylation levels of cancer-driving signaling proteins.

Our findings showed that mutant NRAS/KRAS MM cells were highly sensitive to MEK inhibition, as compared to wild-type (WT) MM cells. Furthermore, a systematic correlation analysis of drug sensitivities and signaling protein readouts revealed several drug dependencies. Overall, both mutational status and protein phosphorylation/expression status may help elucidate drug-specific sensitivities in a small panel of MM cell lines, which could next guide future studies to predict biomarkers of drug sensitivity in the context of MM.

## Materials and methods

### Cell lines

Myeloma cell lines used in this study were U266 (17), JJN3 (18), CAG (19), INA6 (20), OH2 (21, 22), IH1 (23), KJON, VOLIN (24), URVIN, and FOLE. The FOLE MM cell line (Misund et al., unpublished), has biallelically-inactive *TP53*, and was used as a positive control for testing of the MDM2-P53 interaction inhibitor nutlin-3A. The MM cell lines were cultured in RPMI 1640 medium (ThermoFisher Scientific, Waltham, MA, USA) supplemented with 2mM L-glutamine, 1% Penicillin-Streptomycin, 1x Sodium Pyruvate (NaPur), and fetal bovine serum (FBS) at 10% (JJN3, CAG, INA6, VOLIN, URVIN) or 15% FBS (U266), or 10% human serum (Sigma-Aldrich, Saint-Louis, MO, USA) (IH1, OH2, KJON). The culture medium was supplemented with 2 ng/mL IL6 (ThermoFisher Scientific) for culturing of INA6, KJON, IH1, OH2, URVIN, and VOLIN. The cells were expanded, aliquoted and cryopreserved until experimental assays were performed. See **Table 1** for MM cell line characteristics.

**Table 1:** Characteristics of the MM cell lines included in the study.

Cell line	IL-6 dependent	IgH translocations	TP53 status	Other features	High-risk	Ploidy
INA6	yes	t (11, 14)	*TP53* MUT	Up-regulation of *Cyclin D1*	yes	NHRD
JJN3	no	t (14, 16), t (8, 14)	*TP53* missing (1)		yes	NHRD
CAG	no	t (14, 16)	*TP53* MUT		yes	NHRD
U266	no	t (11, 14)		Up-regulation of *Cyclin D1*; Active *STAT3* (2)	no	NHRD
URVIN	yes	t (4, 14)	*TP53* WT	del(1p); gain(1q)	yes	NHRD
IH1	yes	t (4, 14)	*TP53* WT		yes	NHRD
VOLIN **(** 3 **)**	yes	not detected (3)	*TP53* MUT (3)	gain(1q)	yes	HRD
KJON **(** 3 **)**	yes	not detected (3)	*TP53* MUT (3)	del(1p13); gain(1q)	yes	HRD
OH2	yes	unknown	*TP53* WT		yes	HRD
FOLE (unpublished)	no	t (4, 14)	*TP53* MUT (p.P72R; p.C238W) (unpublished)	del (17)	yes	NHRD

HRD, hyperdiploid; NHRD, non-hyperdiploid.

### Cell viability assay

Drugs (n=33) were selected based on mutated targets in the cell lines (see **Tables 2**, **3** for drugs and their corresponding mutated targets), and were added to 384-well TC-microplates (Greiner #781080) using an acoustic dispenser (Echo 550, Labcyte Inc., CA, USA). Each drug was tested at five concentrations ranging from 1 to 10,000 nM. Experiments on cell lines were done on freshly thawed cells. MM cells (5000 cells per well in 25 ul volume) were transferred into plates using an automatic dispenser (Certus Flex, Fitz Gyger, Thun, Switzerland), and incubated at 37°C for 72h. Cell viability was assessed by the CellTiter-Glo luminescence assay (Promega, WI, USA) according to the manufacturer’s instructions. Luminescence was recorded with an Envision Xcite plate reader (Perkin Elmer, MA, USA). The raw concentration-response data were analyzed using the KNIME software (AG, Zurich, Switzerland) and Rstudio Team (Boston, MA) (25). Normalization of the response readout was done to the negative (0.1% DMSO) and positive (100 µM benzethonium chloride) controls.

**Table 2:** MM cell lines included in the study and amino acid changes detected in mutated genes.

Mutated gene	MM cell lines with amino acid changes
*EGFR*	**KJON** (*V592I,R832C*), **OH2** (*Q61K*)
*ABL1*	**IH1** (*H929N*)
*FLT1*	**CAG** (*I178S*), **JJN3** (*E910K*)
*FLT3*	**INA6** (*T526M*)
*FLT4*	**OH2** (*V750M*),**U266** (*G1328V*)
*NRAS*	**IH1** (*G12V*), **INA6** (*G12D*), **JJN3** (*G13D*), **OH2** (*Q61K*)
*KRAS*	**KJON** (*Q61H*)
*ALK*	**JJN3** (*P1213S*)
*ROS1*	**U266** (*S371P*), **URVIN** (*R1569W*)
*SYK*	**IH1**(*D410Y*)
*ALK*	**JJN3** (*P1213S)*
*PIK3CA*	**CAG** (*H1047R*), **JJN3** (*W590C*)
*PIK3R3*	**URVIN** (*X462Y-stop lost*)
*MET*	**KJON** (*V121I)*
*BRAF*	**U266** (*K601N*)
*RAF1*	**CAG** (S259F)
*CAMK*	**KJON** (*Q128K, P342L,P363L*), **CAG** (*V436I,V459I,V445I,V480I,V468I,V497I*)
*CDK13*	**KJON** (*V736E*), **URVIN** (*G65R*),
*CLK1*	**JJN3** (*N99S*), **URVIN** (Q455E)
*MTOR*	**JJN3** (*A2300D*)
*TP53*	**CAG** (E285K), **INA6** (K132M), **VOLIN** (L130V), **U266** (A161T)

The table indicates the names of the “druggable” mutated target gene detected in the indicated MM cell lines (cell line names in bold), the corresponding amino acid substitutions, and for which cell line the mutations occur.

**Table 3:** Drugs included in the study and their associated target.

Drug name	Matched mutated target	Clinical Trials.gov ID*/Reference/Cancer
Afatinib	*EGFR*	NCT02465060 (lymphomas, MM)
AT9283	*ABL1*	(1)
Axitinib	*FLT1/3/4*	NCT03297606 (lymphomas, MM)
binimetinib	*NRAS, KRAS*	NCT02465060 (lymphomas, MM)
Brigatinib	*ALK, ROS1*	NCT02094573; NCT03535740
Cerdulatinib	*SYK*	NCT01994382, NCT04021082 (CLL, lymphomas)
Ceritinib	*ALK*	NCT02393625
Cobimetinib	*NRAS, KRAS*	NCT02465060 (lymphomas, MM)
Copanlisib	*PIK3CA, PIK3R3*	NCT02465060 (lymphomas, MM)
Crizotinib	*ALK, ROS1, MET*	NCT02693535 (MM); NCT04439253; NCT01121588; NCT02465060 (lymphomas, MM)
Dabrafenib	*BRAF*	NCT02465060 (lymphomas, MM); NCT03091257
Dasatinib	*ABL1*	NCT03595917 (leukemia); NCT03297606 (lymphomas, MM)
Entospletinib	*SYK*	NCT01799889 (CLL, lymphomas)
Entrectinib	*ALK, ROS1*	NCT02693535 (lymphomas, MM)
Erlotinib	*EGFR*	NCT03297606 (lymphomas, MM)
GSK2636771	*PIK3CA, PIK3R3*	NCT02465060 (lymphomas, MM)
Idelalisib	*PIK3CA, PIK3R3*	NCT01539512 (CLL)
KN-93 phosphate	*CAMK*	(2, 3)
lorlatinib	*ALK, ROS1*	NCT03052608; NCT03909971; NCT01970865
ML167	*CDK13, CLK1*	(4)
Nutlin-3A	*Mdm2-TP53 (TP53 wild-type)*	
Osimertinib	*EGFR*	NCT02465060 (lymphomas, MM)
palbociclib	*CDK13*	NCT02465060; NCT02693535; NCT03297606 (lymphomas, MM)
pictilisib	*PIK3CA, PIK3R3*	(5)
regorafenib	*FLT1,3,4*	NCT02693535 (lymphomas, MM)
ruxolitinib	*JAK/STAT*	(6)
sunitinib	*FLT3 (VGFR)*	NCT02693535; NCT02465060; NCT03297606 (lymphomas, MM)
TAK-659	*SYK*	(7)
Taselisib	*PIK3CA, PIK3R3*	NCT02465060; NCT04439175 (lymphomas, MM)
Temsirolimus	*MTOR, PIK3CA, PIK3R3*	NCT03297606 (MM), NCT00398515 (MM), NCT00079456 (MM), NCT02693535 (MM)
Trametinib	*NRAS, KRAS*	NCT02642042, NCT03091257 (MM), NCT04487106 (leukemia)
U0126	*NRAS, KRAS*	
Vemurafenib	*BRAF*	NCT03297606; NCT02693535 (lymphomas, MM)

The table contains information about the drugs (n=33) selected in this study, including their target, clinical trial, and if the drug is being tested in MM or related B-cell malignancies.

### Phospho flow

Phospho flow assays were performed on freshly thawed myeloma cells as described previously (26). Antibody-stained samples were run on a BD LSR Fortessa and output data were analyzed in Cytobank (https://cellmass.cytobank.org/cytobank/). Raw data were transformed to an arcsinh ratio relative to the signal of an isotype control, which was set to zero.

### Data analysis and statistics

Curve-fits of normalized concentration-response data used the function drm from the R package drc (https://www.r-project.org/) with the four parameter log-logistic model, LL.4, or the logistic model, L.4, where LL.4 failed to converge. To quantify drug responses, a modified drug sensitivity score (DSS) was calculated for each drug (27). In this modified function, area under the curve was calculated using a response-window from 100% to 10%, and a concentration-window from the minimum concentration tested to the concentration where the viability reached 10% (threshold %). DSS type 1 was used, without the term for division by the logarithm of the upper limit. The DSS scores were calculated on a scale of 0-100. A high DSS therefore indicates that MM cells are drug-sensitive, while a low DSS indicates drug-resistant MM cells.

**Figure 1:**
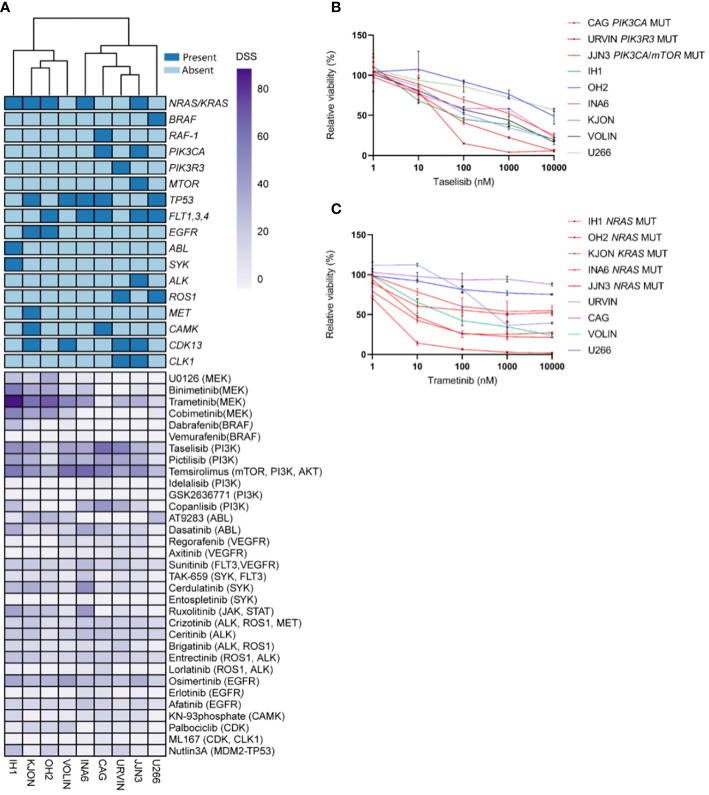
Targeting point mutations in cancer-driving genes in MM cell line models. A–C, Freshly thawed cells from the indicated MM cell lines were dispensed in 384-well plates pre-coated with a customized drug library of 33 single drugs. At 72h, cell viability was assessed by CellTiter-Glo. A drug sensitivity score (DSS) was calculated ranging from 0 to 100 for the entire drug library (see Materials and Methods). High DSS indicates high drug sensitivity. **(A)**, MM cell lines (n=9) were exposed to single drugs as described above. DSS (rows) was calculated for each drug and cell line and plotted as a heatmap. Columns are clustered using Euclidean distance and the Ward linkage method. Column annotations (top) indicate mutated genes, where blue means mutation present, while light blue absent. **(B, C)**, Concentration-response curves for the effects of taselisib and trametinib, on the viability of indicated MM cell lines (72h) treated as above. Graphs show mean viability ± standard deviation (SD, n=3). MM cell lines with mutations in *PI3K*/*mTOR* and *RAS* target genes are indicated in red, whereas the MM cell lines in different colored lines are wild-type and each line indicates cell viability effects in each individual cell line.

Statistical analyses of output data from drug sensitivity screens and phospho flow assays were performed in GraphPad Prism v8 (San Diego, CA, USA). Welch’s t test [(*p < 0.05, ***p < 0.001, ns (not significant)] was used to compare two means, as indicated in the respective figure legends. ClustVis (28) was used for unsupervised clustering of the DSS values and column annotations indicating genes with the presence of point mutations. No scaling is applied to rows. Columns were clustered using Euclidean distance and Ward linkage. The significance of the correlation coefficients between DSS values and phospho flow readouts was assessed by Pearson’s r test for **Figures 1B, C** and **Supplementary Figure 2**, where the p-values prior to multiple-testing correction are shown.

Predictive models for drug response (as defined by the DSS) were constructed using either mutation or phospho flow data as covariates. In each case, a type of multi-response penalized linear regression model known as tree-lasso (29, 30) was used for feature selection and to assess the predictive power of the selected features for drug response. Regression coefficients for all 33 drugs were penalized jointly in a hierarchical fashion, with penalties weighted by the strengths of the correlations among drug responses, as defined by the height parameters in the hierarchical tree structure of the DSS data (**Supplementary Figure 1**). For optimal results, the tree height parameters were cut-off at 0.7, on a scale where the root of the tree is at a height of 1 and the leaves are at a height of zero.

**Figure 2:**
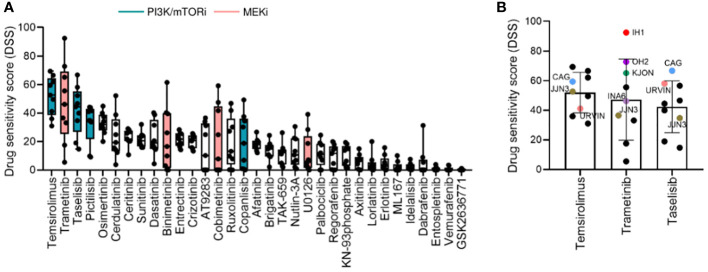
Drug sensitivity profile in MM cell lines. **(A)**, Drug sensitivity to the indicated drugs across the 9 MM cell lines shown in **Figure 1**. The graph shows DSS for the indicated drugs sorted from most to least effective as mean ± SD. Green and pink bars indicate PI3K/mTOR inhibitors (PI3K/mTORi) and MEK inhibitors (MEKi), respectively. **(B)**, DSS distribution for the three most effective drugs (target indicated): temsirolimus (mTOR, PI3K, AKT), trametinib (MEK1/*2*), and taselisib (PI3K) inhibitors in the MM cell lines. MM cell lines with targeted mutations are indicated in different colors.

Both the mutation-dependent and protein-dependent models were trained using leave-one-out cross validation to compute the Mean Squared Error (MSE) as a function of the tuning parameter. The tree-lasso cost function was optimized using the sub-function *tree.lasso* from the R package IPFStructPenalty, available at https://github.com/zhizuio/IPFStructPenalty. In each model, the tuning parameter Λ was set to a value at which the cross-validated MSE becomes essentially flat and the smoothing parameter for Smoothing Proximal Gradient descent (SPG) optimization was set to μ = 10^-4^. For the mutation-dependent model, the tuning parameter was set to Λ = 25 (with a cross-validated MSE of 175), and for the protein-dependent model it was set to Λ = 24 (with a cross-validated MSE of 530). The tree-lasso regression coefficients of each model were then computed using the optimal values of the tuning parameter Λ (**Figures 2A**, **1A, B**).

The amount of variance explained by each model was also assessed by the R squared value, which was computed for each tuned model as R^2^ = 1 - RSS/TSS, where the Residual Sum of Squares (RSS) and Total Sum of Squares (TSS) were both defined using all 9 cell lines at once. The R squared value was R^2^ = 0.39 for the mutation-dependent model, and R^2^ = 0.58 for the protein-dependent model.

The covariates for the protein-dependent model were defined as the standardized phospho flow data, i.e., for each protein the phospho flow arcsinh ratios were centralized at their mean value across samples and normalized by their standard deviation prior to the tree-lasso model calculations. As a consistency check, an overall matching between the signs of the regression coefficients and those of the protein-drug Pearson correlation coefficients was observed (**Supplementary Figure 1B**), since the standardization of the phospho flow data allows analogous interpretations for these two types of coefficients.

### Targeted high-throughput sequencing analysis – variant calling

Targeted DNA sequencing was performed using the SureSelect Human Kinome kit (Agilent Technologies), with capture probes targeting 3.2 Mb of the human genome, including exons and untranslated regions (UTRs) of all known kinases and selected cancer‐related genes (n = 612). Paired-end sequencing reads of 100-bp length were aligned to the human reference genome (hg19) with Novoalign (version 2.08.3), followed by filtering and realignment with GATK tools and Picard. Single point mutations were identified using MuTect (version 1.1.4). Variant consequence annotation was performed with ANNOVAR, using RefSeq as the underlying transcript model. Details of the complete variant calling pipeline that was applied on the MM cell lines have been described previously (31). The mean sequencing coverage of the kinome was 477x, and we obtained a minimum coverage of 150x for 87.6% (VOLIN) and 90.0% (KJON-1) of the kinome targeted regions. Variant calling performed on cancer cell lines without utilizing matched normal samples is bound to generate a mix of germline variants and somatic variants. We therefore set up a set of filtering procedures to both *i)* exclude known germline variants, and *ii)* enrich for coding, cancer-associated variants. Specifically, we excluded all variants that overlapped with germline variants found in the 1000 Genomes Project phase 3 (minor allele frequency > 1% in any population), and NHLBI Exome Sequencing Project (minor allele frequency > 0.1% in any population) (32, 33). In addition, we excluded variants present in the single nucleotide polymorphism database (dbSNP) (build 138) (34) that had no clinical associations (as given from ClinVar cross-references). Finally, we restricted the variant set to coding variants (missense, stop-gain/stop-loss, frameshift/non-frameshift, splice site donor/acceptor) in known cancer census genes (COSMIC version 68) (35). All variants were subjected to a functional annotation workflow that included UniProt KB (functional protein properties) and Polymorphism Phenotyping v2 (PolyPhen-2) web server (computational predictions of effect of coding variants) (http://genetics.bwh.harvard.edu/pph2/) (36).

## Results

### Identification of mutations in MM cell lines

In order to identify functionally relevant and actionable mutations in MM, nine MM cell lines (**Table 1**) were subjected to targeted high-throughput DNA sequencing. Short sequencing reads were processed with a variant calling pipeline and subsequent variant filtering procedure, from which we detected a total of 136 mutated genes in the cell lines (**Supplementary Table 1**). Mutated genes included *NRAS*/*KRAS*, *BRAF*, *RAF-1*, *TP53*, *PIK3CA*, *PIK3R3*, *MTOR*, *FLT1*, *FLT3*, *FLT4*, *EGFR*, and *SYK*, which are known to be frequently mutated in MM and other related blood malignancies (37–39), as well as to have a therapeutic potential in MM (39) (**Tables 2**, **3**).

To prioritize the identified gene variants for functional impact, all protein-coding alterations were subjected to analysis with PolyPhen2 (see Materials and Methods), a tool that provides computational predictions for the functional impact of amino acid changes. We identified 31 amino acid changes with predicted damaging effects which were druggable (39), including the most oncogenic *RAS* mutated isoform, G12 and Q61, as well as *PIK3CA* catalytic subunit mutations, p.H1047R and p.W590C (40, 41) (**Tables 2**, **3** and **Supplementary Table 1**).

### Targeting the mutational status in MM cell lines

Based on the mutation analysis, we designed a drug library consisting of 33 clinically relevant drugs targeting identified druggable gene products (**Table 3**). Drug sensitivity screens were then performed on the 9 MM cell lines.

An unsupervised clustering of the cell lines by their DSS profiles showed that MM cell lines with *PI3K* and *NRAS*/*KRAS* mutations displayed high sensitivity to MEK and PI3K inhibitors, respectively (**Figure 1A**). Concentration-response effects on the MM cell viability to individual inhibitors with and without associated targets are shown in **Figures 1B, C**. These findings support our hypothesis that the presence of *RAS* and *PI3K* mutations may increase MM cell sensitivity to MEK and PI3K inhibitors.

We observed variability in efficacy among drugs targeting the PI3K signaling pathway. The most effective drugs were temsirolimus (mean DSS ± SD = 51.96 ± 13.66), taselisib (mean DSS ± SD = 41.82 ± 17.10), pictilisib (mean DSS ± SD = 32.50 ± 13.66), and copanlisib (mean DSS = 19.83 ± 18.65), while treatment with idelalisib and GSK2636771 resulted in markedly low DSS (mean DSS = 2.29 ± 2.5, mean DSS = 0.26 ± 0.46, respectively) (**Figure 2A**). Among the MEK inhibitors, trametinib induced the highest response (mean DSS ± SD = 47.20 ± 27.46), followed by binimetinib (mean DSS ± SD = 21.88 ± 21.54), cobimetinib (mean DSS ± SD = 21.11 ± 23.80), and U0126 (mean DSS ± SD = 11.83 ± 13.85) (**Figure 2A**).

Interestingly, the MM cell lines CAG and URVIN, harboring *PIK3CA* and *PIK3R3* mutations, respectively, exhibited the highest sensitivity to taselisib and temsirolimus treatment, while JJN3, harboring mutations in *PIK3CA* and *mTOR* genes, showed less sensitivity to taselisib compared to CAG and URVIN cell lines. On the other hand, JJN3 gained sensitivity to temsirolimus that also target *mTOR* (**Figure 2B**). The MM cell lines IH1, OH2, and KJON, with *NRAS*/*KRAS* mutations, were found highly sensitive to trametinib (**Figure 2B**). These results indicate that sensitivity to kinase inhibitors may be associated with corresponding pathway mutations.

### Mutational status as a predictor for *in vitro* drug responsiveness

**Figure 3:**
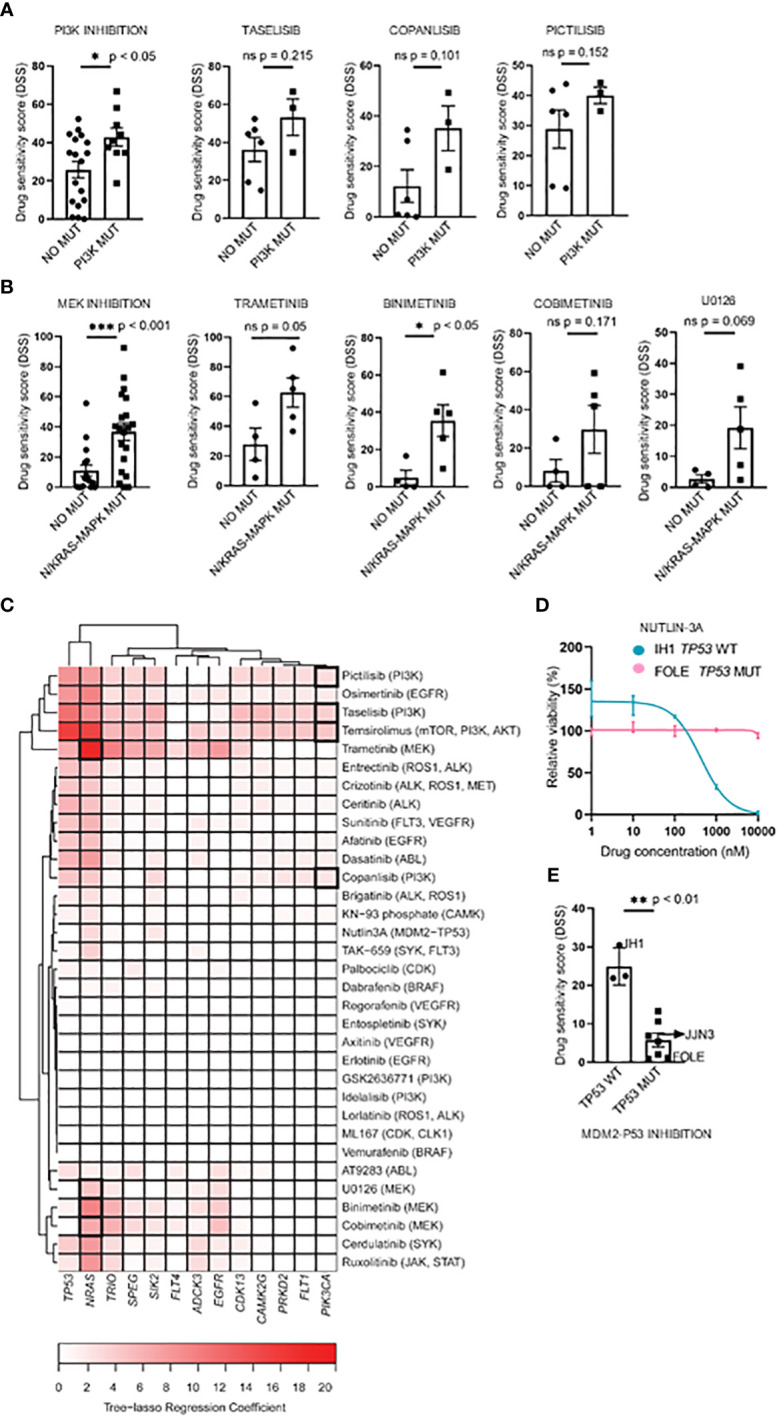
Associations between drug sensitivity and mutational profiles in MM cell lines. **(A, B)**, Pharmacogenomic comparison between *PI3K* and *RAS* mutation status and DSS to PI3K and MEK inhibitors. Aggregated (left) and individual drug (middle-left) effects are shown. The graph shows mean DSS ± SD. **(C)**, Heatmap of the tree-lasso regression coefficients for DSS as a function of mutations, where the rows are all the possible selected features (i.e., genes whose regression coefficients are exactly zero for all drugs were excluded from the plot, as well as those coefficients that, for every drug, are lower in absolute value than 5% of the sum of all coefficients for the given drug). Framed rectangles highlight the predictive power of *NRAS* and *PIK3CA* for the responses to MEKi and PI3Ki, respectively. **(D)**, Concentration-response curves of cell viability for nutlin-3A in the IH1 and FOLE MM cell lines that differ in TP53 mutational status. The graph shows mean viability ± SD (n=3). **(E)**, Association of the *TP53* mutation status with nutlin-3A sensitivity for all 10 MM cell lines. The graph shows mean DSS ± SD. Statistics were performed using Welch’s test. Statistics were performed using Welch’s test. *p < 0.05, **p < 0.01, ***p < 0.001, ns (not significant).

To study whether the mutational status could predict drug sensitivity, we compared treatment responses to PI3K and MEK inhibitors between cell lines with and without selected mutations. We found that cell lines with *PI3K* pathway mutations (i.e. *PIK3CA*, *PIK3R3*, and *mTOR*) or *RAS* gene mutations (i.e. p.G12V, p.G12D, p.G13D, p.Q61K, and p.Q61H) (**Table 2**) exhibited significantly higher DSS relative to cell lines with WT forms of the listed genes when comparing aggregated data for each class of drugs tested (**Figures 3A, B**, left panels). The three PI3K inhibitors, taselisib, copanlisib, and pictilisib, each showed a trend towards association with PI3K mutational status, but this was not statistically significant (**Figure 3A**, right panels). Of the four MEK inhibitors tested, a statistically significant association was found between the response to binimetinib and RAS mutational status whereas trends towards statistical significance were observed for trametinib, cobimetinib, and U0126 (**Figure 3B**, right panels).

Interestingly, by applying a tree-lasso regression model as described in Materials and Methods, a few mutations were found to be associated in varying degrees with high sensitivity to several drugs, as indicated by the positive values in the sparse matrix of regression coefficients (**Figure 3C**). In particular, the mutational status of the *PIK3CA* and *NRAS* genes were selected as predictors for drug sensitivity to PI3K and MEK inhibitors (**Figure 3C**).

We also noticed that, while the prediction analyses demonstrated that the mutational status may be useful to predict drug sensitivity, the predictive impact of *NRAS* mutations on the drug sensitivity to MEK inhibitors was higher than the one observed for *PIK3CA* mutations on PI3K inhibitors, as indicated by the color-intensity of the heatmap in **Figure 3C**.

The inhibition of the MDM2-TP53 interaction by nutlin-3A is an attractive strategy to stabilize the P53 mediated apoptosis in various WT TP53 tumors, including MM (42–44), and therefore worth investigating. Interestingly, we observed differing sensitivities to the MDM2-TP53 inhibitor nutlin-3A between the TP53 WT IH1 and the biallelically TP53 mutant MM cell line FOLE. (**Figure 3D**). When stratifying nutlin-3A responses on TP53 mutational status in all MM cell lines tested, we observed a significantly higher drug sensitivity in the TP53 WT cell lines, in agreement with earlier reports (14, 15, 45) (**Figure 3E**).

### Protein expression level affects drug sensitivities

Having demonstrated that gene mutations in MM cell lines are linked to drug sensitivities towards PI3K and MEK inhibitors, we were then interested in studying how expression levels of the pathway (phospho) proteins correlated with their drug sensitivity.

**Figure 4:**
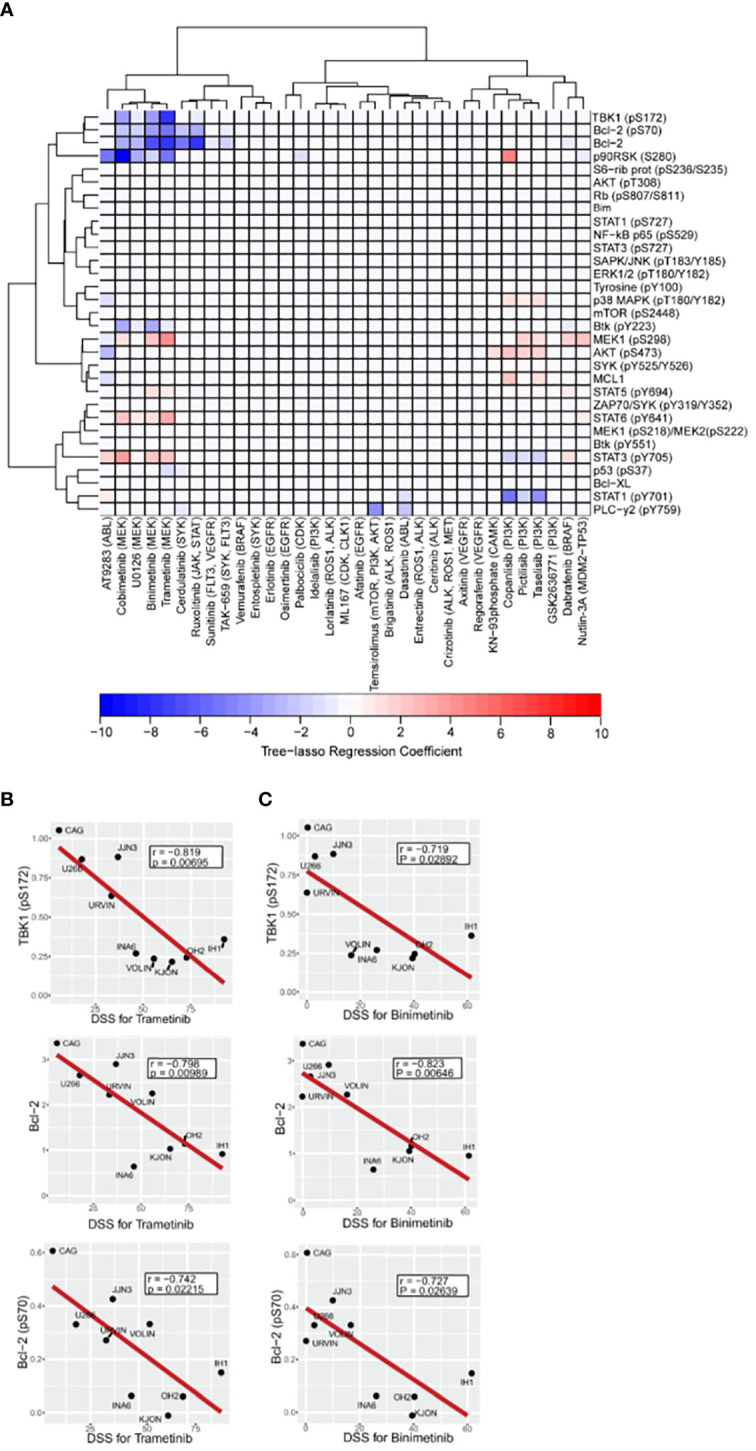
Association between drug sensitivity and expression or activation profile of selected intracellular cancer-driven proteins. A–C, Freshly thawed MM cell lines (n=9) were fixed, permeabilized and stained with antibodies against the indicated proteins or phosphoprotein epitopes (rows). Signals were analyzed by flow cytometry (see Materials and Methods). **(A)**, Heatmap of the tree-lasso regression coefficients for DSS as a linear function of the standardized phospho flow data, with proteins as rows and drugs as columns. Positive and negative regression coefficients are represented in shades of red and blue, respectively. B,C, Pearson correlation plots for DSS to the indicated MEK inhibitors trametinib **(B)** and binimetinib **(C)** versus three signaling readouts inversely associated with drug response.

In order to identify relevant signaling proteins whose observed basal expression or phosphorylation levels across different cell lines can explain corresponding DSS values for different drugs, our first approach was a correlation analysis using Pearson correlation coefficients. Correlation coefficients for all possible protein-drug pairs, including the 33 drugs in our library, and the 31 relevant signaling proteins (16) selected for our study, were computed using the phospho flow readouts and DSS values from all the MM cell lines (**Supplementary Figure 1B**). Multiple significance tests of the Pearson correlation coefficients followed by a false discovery rate controlling procedure revealed that this method is not sufficiently robust to capture significant correlations. Our solution was to model all the drugs jointly using the tree-lasso regression approach described in Materials and Methods. By penalizing regression coefficients jointly according to the hierarchical clustering tree of the correlations among DSS values of different drugs, we were able to achieve enough sensitivity to capture protein-drug correlations that had been lost in our previous, naïve approach (**Figure 4A**). We found that PI3K and MEK inhibitors emerged as separate clusters, each with a common set of predictive variables, as indicated by a few non-zero tree-lasso regression coefficients (**Figure 4A**). We note that since the covariates in our model were defined as the standardized phospho flow readouts, the tree-lasso regression coefficients have a similar interpretation to correlation coefficients (i.e., positive or negative values for the coefficients have similar meanings in both cases), and therefore can be regarded as a special type of correlation.

Notably, using the tree-lasso regression model we found that high levels of phospho-TBK1 inversely correlated with low drug sensitivity to MEK inhibitors, including trametinib, binimetinib, and cobimetinib (**Figure 4A**). Although these correlations seem to be in agreement with individual Pearson correlation plots of the TBK1 levels against DSS for trametinib and binimetinib (upper plots in **Figures 4B, C**), we emphasize that their true significance is only revealed by an integrated model that explores the data structure of all the drugs to define a new, more robust, type of correlation.

As also observed in our previous study (16), we found that high levels of Bcl-2 and phospho-Bcl-2 predicted low sensitivity to trametinib, binimetinib, and cobimetinib (**Figures 4A-C** middle and lower plots). Once again, individual Pearson correlation plots (**Figures 4B, C** middle and lower plots) are not enough to identify these relationships, since their statistical significance were only seen after using the more robust tree-lasso regression approach for variable selection. Also, worth mentioning is our finding that phospho-MEK1 (pS298) is positively correlated (in the sense of positive tree-lasso regression coefficients) with sensitivity to the same MEK inhibitors (trametinib, binimetinib, and cobimetinib). Moreover, we observed that high phosphorylation levels of AKT (pS473) correlated with high responses to PI3K inhibitors taselisib, pictilisib and copanlisib (**Figure 4A**). In this case, Pearson correlation p-values are too high even before multiple-testing correction (**Supplementary Figures 2A-D**), however, tree-lasso regression was still sensitive enough to capture a relatively weak correlation.

Taken together, our results indicate that the expression and phosphorylation levels of signaling proteins can inform on drug sensitivity, and therefore, can provide relevant information as part of functional drug testing studies.

## Discussion

Currently, the choice of therapy for MM patients is mostly based on physician’s decision and patient’s clinical status, including age and comorbidities (46, 47). Identification of molecular drivers and biomarkers is therefore needed to improve the outcome of individual patients. Mutated cancer-driver genes are now employed as drug sensitivity biomarkers in clinical trials for MM, leukemia and lymphomas (48–50) (NCT04470947). Moving forward in this context, the EXALT study (NCT03096821) has demonstrated that drug-response testing in cancer cells combined with molecular profiling data is feasible and can improve treatment outcome (51), providing proof-of-concept data for this approach.

Here, we aimed to test 33 targeted drugs and to compare their sensitivity and relationships to mutational status of selected genes in a panel of MM cell lines. We also performed a comprehensive analysis correlating drug sensitivities to basal expression and phosphorylation levels of signaling proteins to explore the impact of activated mitogenic pathways on differential drug responses.

We found that RAS- and PI3K- related mutations supported MM cell sensitivity to MEK and PI3K inhibitors, respectively. We observed drug-class response differences in drugs with the same target. The PI3K pathway inhibitors temsirolimus, taselisib, pictilisib, and copanlisib were very active, whereas idelalisib and GSK2636771 showed little or no activity in this study. This might be due to isoform-specific effects of the PI3K inhibitors tested, as previously reviewed (52, 53). In addition, the impact of *PIK3CA* mutations on the responses to PI3K inhibitors was moderate compared to that observed for *NRAS* mutations on the effect of MEK inhibitors. However, the observations and prediction model might be impacted by the lower frequency of MM cell lines harboring PIK3CA mutations (2/9) compared to the corresponding frequency for NRAS mutations (4/9). Nevertheless, our data are in agreement with results from a meta-analysis case in breast cancer, where the predictive role of *PIK3CA* mutation status on the clinical efficacy of PI3K inhibitors remains controversial (54).

Trametinib induced the highest sensitivity across the MM cell lines, and sensitivity to both binimetinib and trametinib correlated with the presence of *RAS* mutations, in particular in the *NRAS* isoform. In contrast, we observed that expression or activation of survival members, such as Bcl-2 or phospho-Bcl-2 as well as phospho-TBK1, correlated with low responses to MEK inhibitors (i.e. trametinib, bimetinib).

With respect to *RAS* mutations and prediction of MEK inhibitor sensitivity, the cell line IH1 harboring a p.G12V mutation in *NRAS* had the highest sensitivity to trametinib, followed by the OH2 and KJON cell lines harboring *NRAS* p.Q61K, and *KRAS* p.Q61H, respectively. The presence of *RAS* mutations and RAS pathway activation in lung cancer was previously shown to confer the highest sensitivity to MEK inhibitors among a panel of 500 oncology drugs (55), demonstrating that *RAS* mutations may be indicators of sensitivity to MEK inhibitors.

Of the *RAS* WT cell lines, CAG and U266 show the lowest sensitivity to MEK inhibition by trametinib, whereas URVIN shows reduced sensitivity compared to *RAS* mutant cell lines. In contrast, VOLIN has a sensitivity to trametinib in range with mutant cell lines. This demonstrates that mutational analysis alone is insufficient to predict drug sensitivity, which is in agreement with previous reports (56, 57). Next, we found that the MM cell lines CAG and URVIN with *PIK3CA* and *PIK3R3* mutations were the most sensitive to taselisib-induced inhibition. Taselisib is a selective *PI3Kα* isoform-specific *PI3K* inhibitor. Similarly, a study in *PIK3CA* positive primary uterine carcinomas cells, demonstrated a stronger growth inhibition by taselisib, when compared with WT cells, as well as *in vivo* tumor growth inhibition in mice with *PIK3CA* mutation (58). A phase II study on taselisib is ongoing for patients with *PIK3CA* mutation and advanced refractory solid tumors, lymphomas or MM (NCT02465060). This may indicate that patients with *PIK3CA* mutations may be more addicted to taselisib, and further studies on this are warranted.

Since molecularly targeted therapies act on proteins, measuring the expression and activation at this level is critical to optimize the selection of targeted therapies. We therefore looked at several relevant signaling proteins and their relationships with drug sensitivity. We found that low sensitivity to the *MEK* inhibitors trametinib and binimetinib observed in some MM cell lines, including CAG and U266 with *RAF-1* (*CRAF*) and *BRAF* mutations (see **Figure 1A**), could possibly be explained by high basal expression levels of phospho-TBK1 and Bcl-2 proteins. It has been suggested that another effective strategy to enhance the efficacy of MEK inhibitors involves simultaneous targeting of proteins that are outside the RAS pathway (40). An example is TBK1, an atypical I-κB kinase family member that acts through the Ral guanine exchange factor (RalGEF) cascade to promote tumor signaling, including activation of AKT (59, 60) and NF-κB (61, 62). Targeting TBK1 pharmacologically or by mRNA knockdown induces apoptosis and reduces cell viability in a subset of acute myeloid leukemia (AML) cells with an activated MYC signaling pathway necessary for survival (63). It has been shown that combined TBK1/MEK inhibitors synergistically enhance apoptosis in several model systems with mutated *RAS*, including lung cancer, melanoma cells, as well as in BRAF mutant melanoma cells, resistant to MEK (62, 64–66), warranting further investigation of combined use of TBK1 and MEK inhibitors also in MM.

We found that high basal expression levels of Bcl-2 predict low drug sensitivity to the MEK inhibitors trametinib, binimetinib and cobimetinib. Several pre-clinical and clinical studies have indicated cooperative activity between MEK and Bcl-2 antagonists in solid tumors, MM, and leukemias (67, 68) (NCT03312530), (NCT02670044), (NCT04487106). We have previously also reported synergistic effects of trametinib and venetoclax *in vitro* in chronic lymphocytic leukemia and MM (16). This indicates that our approach can identify markers that affect drug response phenotypes, which may have clinical utility.

In summary, we provide an integrated approach that uses mutational status and profiling of intracellular (phospho) proteins to test how these markers inform on drug sensitivity of targeted treatments. However, the small sample size of the cell lines in this study limited our ability to draw clear conclusions on the predictive value of this approach. The next step will be to improve the prediction significance by including a larger cohort of samples, which will also accommodate for more extensive multiple correlations analyses. While our results suggest that MM cells are sensitive to MEK and PI3K single agents, MM patients are often treated with more than one drug at the same time. Hence it would be of interest to test the efficacy of combined MEK/PI3K inhibition in the future, and also to add conventional agents, including proteasome inhibitors.

## Data availability statement

The datasets presented in this article are not readily available because the data are human identifiable and cannot be openly shared according to Norwegian legislation. Data can only be shared on a collaborative basis based on approval from the Regional Ethics Committee. Requests to access the datasets should be directed to the corresponding author.

## Author contributions

SS and MG designed the research together with KT. MG and SS designed methodology, performed the experiments, analyzed and interpreted data together with KT and LS. LS performed modeling of prediction analysis. TH, SN, EC, KM, AW, EH, DV, and LM-Z provided the MM cell lines with DNA sequencing data, and performed analyses of raw sequencing data. MG wrote the paper with input from KT and SS. All authors read and commented on draft versions of the manuscript and approved the final version.

## Funding

This work was supported by the Regional Health Authority for South-Eastern Norway (2015031), the Research Council of Norway Centre for Digital Life PINpOINT project (294916) and the Stiftelsen Kristian Gerhard Jebsen (grant 19). This project has also received funding from the European Union’s Horizon 2020 Research and Innovation Programme under the Marie Skłodowska-Curie grant agreement (801133), and the Research Council of Norway under the frame of ERA PerMed (322898). MG was a PhD fellow funded by the UiO: Life Science convergence environment PerCaThe when this work was performed. The exome data were produced by the Genomics Core Facility (oslo.genomics.no) as part of the Norwegian Cancer Genomics Consortium cohort.

## Acknowledgments

We thank Martine Schrøder for excellent technical assistance, Thea Kristin Våtsveen for helping with cell culturing and for providing the FOLE cell line, and Alexandra Gade at the Chemical Biology Screening Platform at Centre for Molecular Medicine Norway (NCMM), University of Oslo, for assistance with drug sensitivity screens. We also thank Zhi Zhao at the Oslo Center for Biostatistics and Epidemiology, University of Oslo, for useful discussions and for suggesting his own R implementation of tree-lasso regression (available online at https://github.com/zhizuio/IPFStructPenalty). We would also like to thank Arnoldo Frigessi, from the Oslo Center for Biostatistics and Epidemiology, University of Oslo, for fruitful suggestions that helped to significantly improve the presentation of our results.

## Conflict of interest

The authors declare that the research was conducted in the absence of any commercial or financial relationships that could be construed as a potential conflict of interest.

## Publisher’s note

All claims expressed in this article are solely those of the authors and do not necessarily represent those of their affiliated organizations, or those of the publisher, the editors and the reviewers. Any product that may be evaluated in this article, or claim that may be made by its manufacturer, is not guaranteed or endorsed by the publisher.
