# Supplementary material for: Mutational analysis and protein profiling predict drug sensitivity in multiple myeloma cell lines
Source: Front Oncol. 2022 Nov 29;12:1040730. doi: 10.3389/fonc.2022.1040730 (PMC9745900; doi:10.3389/fonc.2022.1040730)
Supplement: Supplementary file 1 [file DataSheet_1.zip › Supplementary Figure Captions.PDF]

SUPPLEMENT TO

Mutational analysis and protein profiling predict drug sensitivity in multiple myeloma cell lines

Mariaserena Giliberto[1,2], Leonardo Miranda Santana[1,2,3], Toril Holien[4,5,6], Kristine Misund[4], Sigve Nakken[7,8,9], Daniel Vodak[7,8,10], Eivind Hovig[7,8,11], Leonardo A. Meza-Zepeda[7,8,10], Eivind Coward[4,12], Anders Waage[4,5,6], Kjetil Taskén[1,2,*] and Sigrid S Skånland[1,2]

SUPPLEMENTARY FIGURES

Supplementary Figure 1. Tree-lasso regression parametrization and training, and Pearson correlation

A, Hierarchical clustering tree for drug responses (DSS) used for parametrizing the tree-lasso regression penalty weights. The distance metric between drugs *i* and *j* was defined as $dist(i,j) = 1-|r_{ij}|$, where r_{ij} is the Pearson correlation coefficient between the DSS values of *i* and *j*. The complete-linkage method was used for agglomerative hierarchical clustering. The height parameters were normalized such that the root of the tree is at a height of 1 and the leaves are at a height of zero. **B,** Pearson correlation coefficients between DSS in response to the indicated drugs (columns) (data from Figure 1) versus basal expression or phosphorylation level of intracellular proteins. Increasing correlation is indicated in red for positive and in blue for negative.

Supplementary Figure 2. Representative associations between DSS and phosphorylated protein targets

A-D, Correlation plots in MM cell lines (n=9) were done based on the basal expression or phosphorylation level of intracellular proteins and DSS to the indicated inhibitors. The plots show a trend towards a positive correlation to the associated targets.

SUPPLEMENTARY TABLES

Supplementary Table 1. List of mutated genes detected in this study by high-throughput DNA sequencing.

Supplementary Table 2. Computational prediction of damaging effects of mutations detected in the MM cell lines. The table indicates in which gene the genetic substitution occurs. Damaging effects refers to all the listed genetic substitutions. The functional prediction of damaging effects was computed by Polymorphism Phenotyping v2 (PolyPhen-2) web server (see Materials and Methods).
