# Supplementary material for: Mutational analysis and protein profiling predict drug sensitivity in multiple myeloma cell lines
Source: Front Oncol. 2022 Nov 29;12:1040730. doi: 10.3389/fonc.2022.1040730 (PMC9745900; doi:10.3389/fonc.2022.1040730)
Supplement: Supplementary file 1 [file DataSheet_1.zip › Supplementary Table 1.PDF]

Supplementary Table 1. List of Mutations and mutated genes

Cell line	Chr	Pos	Ref	Alt	Gene	amino acid changes	Type
CAG	1	227221075	A	G	*CDC42BPA*	p.V1057A,p.V1138A	missense_variant
CAG	2	112656320	C	T	*MERTK*	p.P3L	missense_variant
CAG	2	171371496	G	A	*MYO3B*	p.E1146K	missense_variant
CAG	3	12645693	G	A	*RAF1*	p.S259F	missense_variant
CAG	3	142281226	C	T	*ATR*	p.D340N	missense_variant
CAG	3	178952085	A	G	*PIK3CA*	p.H1047R	missense_variant
CAG	4	79780782	C	G	*BMP2K*	p.Q310E	missense_variant
CAG	4	79780831	C	G	*BMP2K*	p.S326C	missense_variant
CAG	4	151023668	A	G	*DCLK2*	p.K154E	missense_variant
CAG	6	2683312	C	G	*MYLK4*	p.Q210H	missense_variant
CAG	10	26315456	G	T	*MYO3A*	p.K316N	missense_variant
CAG	10	75574955	C	T	*CAMK2G*	p.V436I,p.V459I,p.V445I,p.V480I,p.V468I,p.V497I	missense_variant
CAG	11	33374898	A	T	*HIPK3*	p.R1144S	missense_variant
CAG	12	14794126	C	T	*GUCY2C*	p.R653H	missense_variant
CAG	13	29008338	A	C	*FLT1*	p.I178S	missense_variant
CAG	17	7577085	C	T	*TP53*	p.E285K	missense_variant
CAG	19	47207621	G	A	*PRKD2*	p.R232C	missense_variant
IH1	1	114499807	G	T	*HIPK1*	p.V552L	missense_variant
IH1	1	115258747	C	A	*NRAS*	p.G12V	missense_variant
IH1	1	179077617	G	T	*ABL2*	p.H929N	missense_variant
IH1	5	14485179	T	G	*TRIO*	p.V2220G	missense_variant
IH1	9	93639899	G	T	*SYK*	p.D410Y	missense_variant
IH1	11	111574079	G	A	*SIK2*	p.G294R	missense_variant
IH1	19	42738703	C	A	*GSK3A*	p.G265V	missense_variant
INA6	1	115258747	C	T	*NRAS*	p.G12D	missense_variant
INA6	1	182554998	G	A	*RNASEL*	p.S315F	missense_variant
INA6	1	204419026	A	T	*PIK3C2B*	p.V729E	missense_variant
INA6	1	227152813	C	T	*ADCK3*	p.S97F	missense_variant
INA6	3	41504688	C	T	*ULK4*	p.V1095M	missense_variant
INA6	3	138433423	T	C	*PIK3CB*	p.M397V	missense_variant
INA6	5	1268689	C	T	*TERT*	p.S843N	missense_variant
INA6	12	120152023	G	T	*CIT*	p.R1387S	missense_variant
INA6	13	28609652	G	A	*FLT3*	p.T526M	missense_variant
INA6	17	7576855	G	A	*TP53*	p.Q331X	stop_gained
INA6	17	7578535	T	A	*TP53*	p.K132M	missense_variant
INA6	17	29554560	C	G	*NF1*	p.A782G	missense_variant
INA6	18	59854839	A	G	*KIAA1468*	p.E34G	missense_variant
INA6	20	9546688	G	T	*PAK7*	p.P445H	missense_variant
JJN3	1	11181337	G	T	*MTOR*	p.A2300D	missense_variant
JJN3	1	22928223	C	T	*EPHA8*	p.R1003W	missense_variant
JJN3	1	114964110	G	T	*TRIM33*	p.S670X	stop_gained
JJN3	1	115258744	C	T	*NRAS*	p.G13D	missense_variant
JJN3	2	29443580	G	A	*ALK*	p.P1213S	missense_variant
JJN3	2	201726055	T	C	*CLK1*	p.N99S	missense_variant
JJN3	3	123813715	C	T	*KALRN*	p.L11F	missense_variant
JJN3	3	178937382	G	T	*PIK3CA*	p.W590C	missense_variant
JJN3	3	195594063	G	T	*TNK2*	p.Q987K	missense_variant
JJN3	4	956276	C	T	*DGKQ*	p.A721T	missense_variant
JJN3	5	149449785	G	T	*CSF1R*	p.P427T	missense_variant
JJN3	7	6085767	C	A	*EIF2AK1*	p.D189Y	missense_variant
JJN3	9	96054921	C	A	*WNK2*	p.A1762D	missense_variant
JJN3	12	40677790	G	T	*LRRK2*	p.Q785H	missense_variant
JJN3	13	28901667	C	T	*FLT1*	p.E910K	missense_variant
JJN3	16	89762172	G	A	*CDK10*	p.E270K	missense_variant
JJN3	17	4795012	G	C	*MINK1*		splice_donor_variant
JJN3	19	40886274	C	A	*HIPK4*	p.D542Y	missense_variant
JJN3	19	47207756	T	A	*PRKD2*	p.E221V	missense_variant
KJON	2	42275643	C	G	*PKDCC*	p.P102A	missense_variant
KJON	2	128087575	A	T	*MAP3K2*	p.L130I	missense_variant
KJON	2	209101880	C	T	*IDH1*	p.D390N	missense_variant
KJON	2	220354391	C	A	*SPEG*	p.P2884Q	missense_variant
KJON	3	49940577	G	T	*MST1R*	p.H156N	missense_variant
KJON	3	96962802	C	A	*EPHA6*	p.P426H	missense_variant
KJON	5	14485179	T	G	*TRIO*	p.V2220G	missense_variant
KJON	5	110782454	C	T	*CAMK4*	p.P177L	missense_variant
KJON	5	149636165	G	T	*CAMK2A*	p.Q128K	missense_variant
KJON	7	40041484	T	A	*CDK13*	p.V736E	missense_variant
KJON	7	55233024	G	A	*EGFR*	p.V592I	missense_variant
KJON	7	55259436	C	T	*EGFR*	p.R832C	missense_variant
KJON	7	116339499	G	A	*MET*	p.V121I	missense_variant
KJON	10	75585071	G	A	*CAMK2G*	p.P342L,p.P363L	missense_variant
KJON	11	113711409	G	C	*USP28*	p.P149A	missense_variant

KJON	12	25380275	T	G	*KRAS*	p.Q61H	missense_variant
KJON	12	121693589	T	C	*CAMKK2*	p.Q272R	missense_variant
KJON	17	7577579	G	T	*TP53*	p.Y234X	stop_gained
KJON	17	21207813	T	G	*MAP2K3*	p.L215W	missense_variant
KJON	19	15374244	G	T	*BRD4*	p.A443D	missense_variant
KJON	X	153283793	T	C	*IRAK1*	p.E248G	missense_variant
OH2	1	115256530	G	T	*NRAS*	p.Q61K	missense_variant
OH2	1	227174346	C	T	*ADCK3*	p.L618F	missense_variant
OH2	5	108294975	T	C	*FER*	p.I528T	missense_variant
OH2	5	180047927	C	T	*FLT4*	p.V750M	missense_variant
OH2	7	55233024	G	A	*EGFR*	p.V592I	missense_variant
OH2	16	2607765	T	C	*PDPK1*	p.V29A	missense_variant
U_266	2	37513440	C	A	*PRKD3*	p.V264L	missense_variant
U_266	3	43344924	C	T	*SNRK*	p.R77C	missense_variant
U_266	3	89521651	A	G	*EPHA3*	p.I910V	missense_variant
U_266	4	48116409	G	C	*TXK*	p.I9M	missense_variant
U_266	4	66233102	C	T	*EPHA5*	p.E633K	missense_variant
U_266	5	146619243	G	T	*STK32A*	p.E16X	stop_gained
U_266	5	180030301	C	A	*FLT4*	p.G1328V	missense_variant
U_266	6	117715378	A	G	*ROS1*	p.S371P	missense_variant
U_266	7	106508148	C	T	*PIK3CG*	p.Q48X	stop_gained
U_266	7	140453132	T	A	*BRAF*	p.K601N	missense_variant
U_266	9	77436584	C	G	*TRPM6*		splice_donor_variant
U_266	13	48934228	A	G	*RB1*	p.K228R	missense_variant
U_266	13	48951156	G	T	*RB1*	p.E440X	stop_gained
U_266	16	22237255	T	C	*EEF2K*	p.Y69H	missense_variant
U_266	16	68842727	T	G	*CDH1*	p.D221E	missense_variant
U_266	17	7578449	C	T	*TP53*	p.A161T	missense_variant
U_266	17	48276647	C	A	*COL1A1*	p.Q137H	missense_variant
U_266	18	56205120	C	T	*ALPK2*	p.D767N	missense_variant
U_266	20	45317948	G	T	*TP53RK*	p.L36M	missense_variant
U_266	22	21083927	G	A	*PI4KA*	p.S1494F,p.S246F	missense_variant
URVIN	1	2105417	A	T	*PRKCZ*	p.D456V	missense_variant
URVIN	1	22920174	A	T	*EPHA8*	p.K533I	missense_variant
URVIN	1	46509345	T	G	*PIK3R3*	p.X462Y	stop_lost
URVIN	2	201718121	G	C	*CLK1*	p.Q455E	missense_variant
URVIN	4	1806119	G	A	*FGFR3*	p.G380R	missense_variant
URVIN	6	3106057	C	T	*RIPK1*	p.H450Y	missense_variant
URVIN	6	117662760	G	A	*ROS1*	p.R1569W	missense_variant
URVIN	7	39990433	G	C	*CDK13*	p.G65R	missense_variant
URVIN	7	143092553	G	A	*EPHA1*	p.Q648X	stop_gained
URVIN	9	35609159	G	A	*TESK1*	p.R434H	missense_variant
URVIN	11	64606221	G	A	*CDC42BPG*	p.R344W	missense_variant
URVIN	11	111593408	A	C	*SIK2*	p.K692T	missense_variant
URVIN	11	111593426	A	C	*SIK2*	p.Q698P	missense_variant
URVIN	12	18466960	C	T	*PIK3C2G*	p.Q367X	stop_gained
URVIN	15	101523786	C	G	*LRRK1*	p.Y105X	stop_gained
URVIN	15	101523788	T	A	*LRRK1*	p.L106Q	missense_variant
URVIN	17	56676452	C	G	*TEX14*	p.E758Q	missense_variant
URVIN	18	18625344	T	C	*ROCK1*	p.N167D	missense_variant
VOLIN	1	45115418	C	A	*RNF220*	p.R511S	missense_variant
VOLIN	1	198231712	A	G	*NEK7*	p.D69G	missense_variant
VOLIN	2	220348261	C	G	*SPEG*	p.R2026G	missense_variant
VOLIN	5	96498899	A	C	*RIOK2*	p.L509V	missense_variant
VOLIN	7	98601827	G	A	*TRRAP*	p.V3428I	missense_variant
VOLIN	8	42147690	G	A	*IKBKB*	p.V73M	missense_variant
VOLIN	10	89720653	C	A	*PTEN*	p.D268E	missense_variant
VOLIN	12	1005356	G	T	*WNK1*	p.E1901D	missense_variant
VOLIN	12	96717807	G	A	*CDK17*	p.P68S	missense_variant
VOLIN	17	7290737	C	T	*TNK1*	p.R485W	missense_variant
VOLIN	17	7578542	G	C	*TP53*	p.L130V	missense_variant
VOLIN	17	74383275	G	A	*SPHK1*	p.E255K	missense_variant
VOLIN	19	49130979	C	A	*SPHK2*	p.P181H	missense_variant
VOLIN	20	30674511	G	A	*HCK*	p.A306T	missense_variant
