# Supplementary material for: Mutational analysis and protein profiling predict drug sensitivity in multiple myeloma cell lines
Source: Front Oncol. 2022 Nov 29;12:1040730. doi: 10.3389/fonc.2022.1040730 (PMC9745900; doi:10.3389/fonc.2022.1040730)
Supplement: Supplementary file 1 [file DataSheet_1.zip › Supplementary Table 2.pdf]

Supplementary Table 2: Computational prediction of damaging effects of mutations detected in the MM cell lines.

Mutated genes	Amino acid change of mutated genes	Functional prediction
EGFR	V592I, R832C, V592I	damaging
ABL1	H929N	damaging
FLT1	I178S, E910K	damaging
FLT3	T526M	damaging
FLT4	V750M, G1328V	damaging
NRAS	G12D, G12V, Q61K	damaging
RAF1	S259F	damaging
ALK	P1213S	damaging
ROS1	S371P, R1569W	damaging
SYK	D410Y	damaging
PIK3CA	H1047R, W590C	damaging
BRAF	K601N	damaging
ABL1	H929N	damaging
EGFR	V592I, R832C, V592I	damaging
CDK13	V736E, G65R	damaging
TP53	E285K, K132M, L130V	damaging
SYK	D410Y	damaging
MTOR	A2300D	damaging
