# Supplementary figures and images for: Mutational analysis and protein profiling predict drug sensitivity in multiple myeloma cell lines
Source: Front Oncol. 2022 Nov 29;12:1040730. doi: 10.3389/fonc.2022.1040730 (PMC9745900; doi:10.3389/fonc.2022.1040730)

Supplementary Figure 1

A

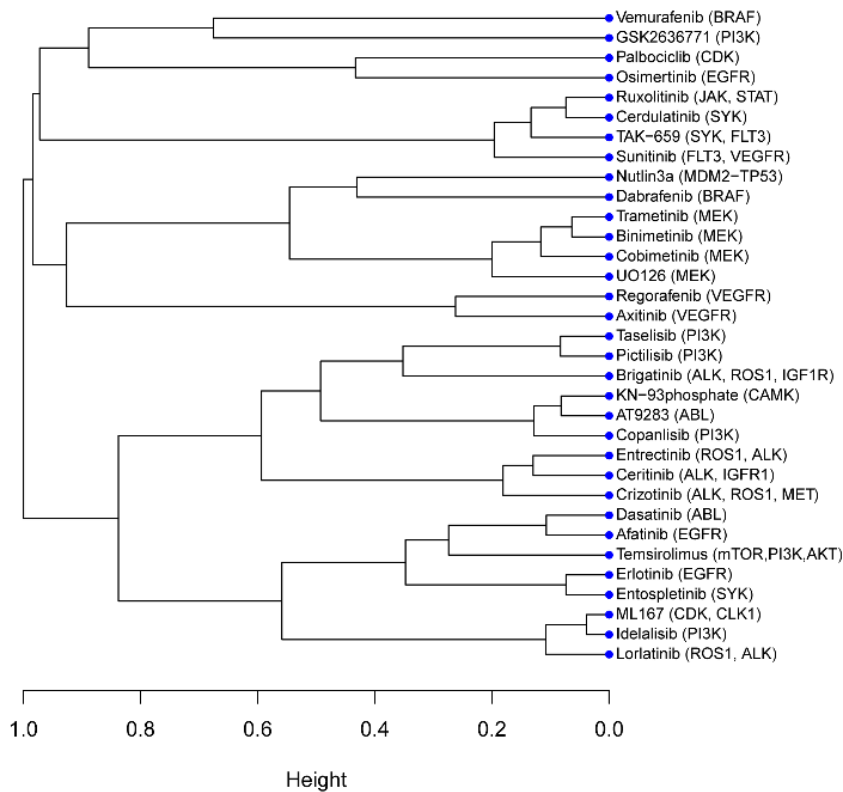

B

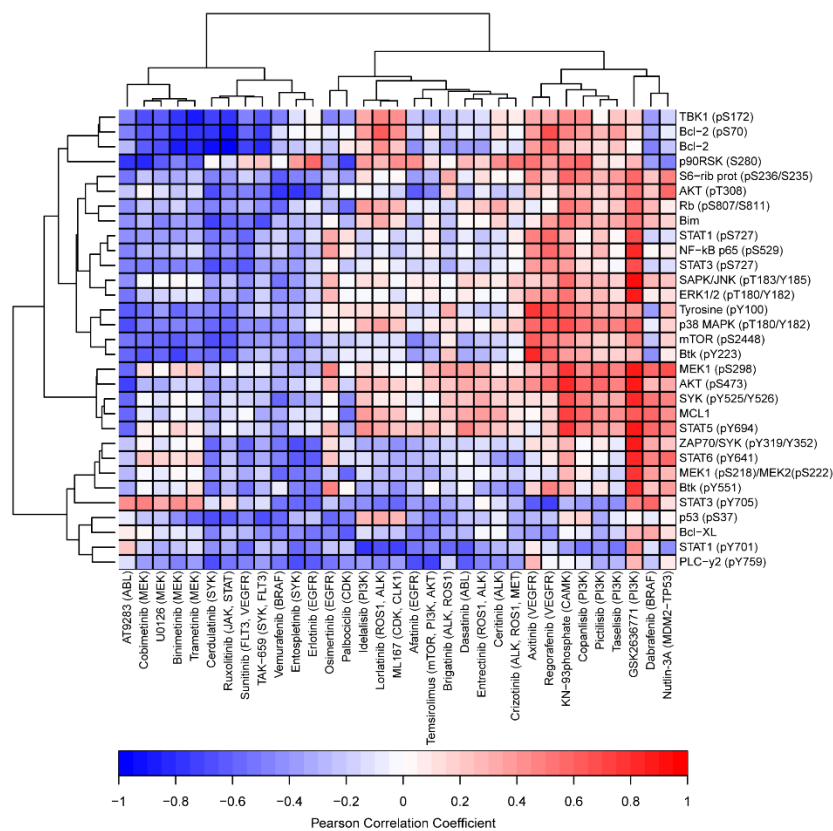

Supplementary Figure 2

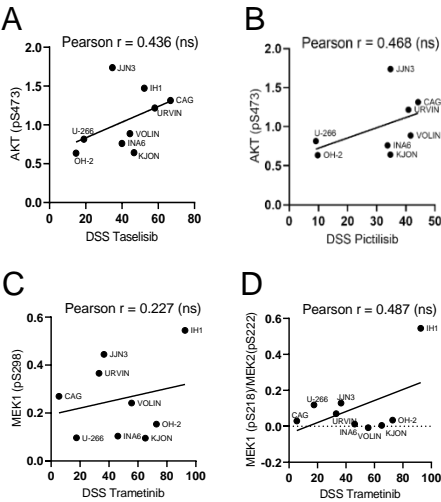

Supplement: Supplementary file 1 [file DataSheet_1.zip › Supplementary Figures.PDF]
